# Sisvan food intake markers from six to 23 months: critical analysis and validation

**DOI:** 10.11606/s1518-8787.2024058005811

**Published:** 2024-07-22

**Authors:** Bianca de Melo Guedes, Thanise Sabrina Souza Santos, Paula dos Santos Leffa, Sara Araújo da Silva, Bárbara Hatzlhoffer Lourenço

**Affiliations:** I Universidade de São Paulo Faculdade de Saúde Pública Programa de Pós-Graduação Nutrição em Saúde Pública São Paulo SP Brasil Universidade de São Paulo. Faculdade de Saúde Pública. Programa de Pós-Graduação Nutrição em Saúde Pública. São Paulo, SP, Brasil; II Universidade de São Paulo Faculdade de Saúde Pública Núcleo de Pesquisas Epidemiológicas em Nutrição e Saúde São Paulo SP Brasil Universidade de São Paulo. Faculdade de Saúde Pública. Núcleo de Pesquisas Epidemiológicas em Nutrição e Saúde. São Paulo, SP, Brasil; III Ministério da Saúde Coordenação de Gestão de Projetos de Pesquisa Brasília DF Brasil Ministério da Saúde. Coordenação de Gestão de Projetos de Pesquisa. Brasília, DF, Brasil; IV Ministério da Saúde Coordenação-Geral de Alimentação e Nutrição Brasília DF Brasil Ministério da Saúde. Coordenação-Geral de Alimentação e Nutrição. Brasília, DF, Brasil; V Universidade de São Paulo Faculdade de Saúde Pública Departamento de Nutrição São Paulo SP Brasil Universidade de São Paulo. Faculdade de Saúde Pública. Departamento de Nutrição. São Paulo, SP, Brasil

**Keywords:** Food and Nutrition Surveillance, Food Intake, Complementary Feeding, Infants, Validation Study

## Abstract

**OBJECTIVE:**

To explore the internal structure and analyze evidence of measurement invariance of the Sisvan form of food intake markers of the Food and Nutrition Surveillance System (Sisvan) for children aged six to 23 months.

**METHODS:**

Sisvan microdata from 2015 to 2019 were used. After analyzing sample adequacy, exploratory factor analysis was carried out considering factor loadings (≥ 0.30 and < 0.85), communality (> 0.20), model fit indices – Tucker-Lewis index (TLI) > 0.90, and root mean square error of approximation (RMSEA) < 0.08. A critical analysis of the form items was performed to adjust the parameters. Measurement stability was investigated between age groups, macro-regions and longitudinally by multi-group confirmatory factor analysis in configural, metric, and scalar invariance models. Configural invariance was accepted if RMSEA < 0.08 and TLI and comparative fit index (CFI) > 0.90. Metric and scalar invariances were accepted if ΔRMSEA < 0.015 and ΔCFI < 0.01, compared to the previous model.

**RESULTS:**

After critical analysis, items were grouped (vegetables and leafy greens; meat or eggs and liver) and excluded (salty food; porridge with milk; rice, potatoes, yams, cassava, flour, or pasta). The exploratory model comprised three factors: healthy complementary feeding (fruit; vegetables; orange or dark green leafy vegetables or fruits; meat, offal, or egg; beans), ultra-processed (yogurt; hamburger and/or sausages; sweetened drinks; instant noodles, packet snacks, or salty cookies; sandwich cookies, sweets, or treats), and milk feeding (breast milk; other milk), with satisfactory factor loadings, communalities and fit (TLI: 0.918, RMSEA: 0.071). The reformulated instrument was stable in the invariance models tested.

**CONCLUSIONS:**

With the incorporation of the modifications to the Sisvan form, the food intake markers subsidize a more adequate interpretation of the feeding situation of children aged six to 23 months.

## INTRODUCTION

The first two years of life are crucial for promoting healthy habits^1^. Between six and 23 months, the start of complementary feeding concomitant with the continuation of breastfeeding is sensitive to the occurrence of inadequate feeding practices^2,3^. According to the United Nations Children’s Fund, the lack of diversity, low food frequency, and consumption of ultra-processed foods in this period present risks for the development of malnutrition^3^.

Demographic and health surveys in 91 low and middle-income countries found that 44.0% of children aged six to 23 months did not eat fruit and vegetables and 42.1% did not eat meat or eggs^4^. Overall, only 27.1% had a diet with the minimum diversity of food groups (95%CI: 23.9-30.3) and 48.7% ate meals at the minimum recommended frequency (95%CI: 45.2-52.2)^5^.

In the Brazilian National Survey on Child Nutrition (Enani-2019),^6,7^ there was a high prevalence of consumption of ultra-processed foods (80.5%) and exposure to sugar (68.4%) from six to 23 months of age, concomitant with the absence of fruit and vegetable intake in 22.2% of children^8^. This highlights the need for systematic monitoring of children’s diets.

In Brazil, food and nutrition surveillance (FNS), within the scope of primary health care (PHC) of the Unified Health System (SUS), includes the assessment of food intake markers. For children aged six to 23 months, the Food and Nutrition Surveillance System (Sisvan) focuses on food quality and the risk of nutritional deficiencies and excess weight^9^.

Although the use of Sisvan food intake markers has been recommended since 2015, there is no evidence of the instrument’s validity, especially regarding its internal structure, in light of dietary recommendations for this age group. In addition, considering how widely the form is used, there is no evidence to support the comparability of information across age groups, given the successive stages of food introduction from six to 23 months, and between the different dietary contexts in the country. The stability of the measurement of the markers over the years, which is relevant to the temporal variations in the population’s dietary practices, is also unknown.

Thus, this study aimed to explore the internal structure of the Sisvan form of food intake markers for children aged six to 23 months and analyze evidence of measurement invariance between age groups, Brazilian macro-regions and longitudinally, from 2015 to 2019.

## METHODS

### Sisvan Food Intake Markers for Children aged Six to 23 Months and 29 Days

The Sisvan form of food intake markers for children aged six to 23 months and 29 days includes 20 questions referring to the previous day^9^. For the present study, 17 items on the consumption of foods and/or food groups were considered: “breast milk”, “whole fruit, in pieces or mashed”, “salty food”, “milk other than breast milk”, “porridge with milk”, “yogurt”, “vegetables”, “orange or dark green leafy vegetables or fruits”, “leafy vegetables”, “meat or egg”, “liver”, “beans”, “rice, potatoes, yams, cassava, flour, or pasta”, “hamburgers and/or sausages”, “sweetened drinks”, “instant noodles, packet snacks, or salty cookies”, and “sandwich cookies, sweets, or candies”.

### Data Assignment and Management

The General Coordination of Food and Nutrition (CGAN) provided the Sisvan microdata for the period 2015 to 2019, in accordance with Ministry of Health Ordinance No. 884/2011^10^, and the study was approved by the Research Ethics Committee of the School of Public Health at the University of São Paulo (opinion No. 4.172.787). When managing the data, the records were examined according to: identification number, gender, date of birth, municipality code, and follow-up date, in order to exclude repeated occurrences. In cases of multiple records of the same individual on a single follow-up date (10.35% of observations), the last record of the day was kept. Records of individuals with the same identification number from different municipalities were subtracted (0.44% of observations). Observations with inconsistent age were excluded (0.13% of observations).

For the analyses, only the first record of the individual per year of follow-up was kept, avoiding correlated answers at different points in time. In line with the requirements for psychometric analysis, only records with valid answers (“yes” or “no”) for all items were considered; “don’t know” or blank answers accounted for 9.84% of the records.

The analytical sample comprised 576,034 records of food intake markers for children aged six to 23 months and 29 days between 2015 and 2019. The data was managed using Stata software, version 13.

### Analysis of the Internal Structure of the Sisvan Food Intake Markers for Children Aged Six to 23 Months and 29 Days

Evidence of the validity of an instrument falls into five categories: content, response process, internal structure, relationship with other variables, and consequence of testing^11^. This study focused on analyzing the internal structure of food intake markers, using exploratory factor analysis (EFA). In this interdependence technique, variables are grouped into factors that allow latent evaluation dimensions to be identified according to the correlations between them, without previously defining the structure of the data^12,13^. As a prerequisite for the EFA, the sample adequacy analysis was carried out using Bartlett’s sphericity test, which checks the overall statistical significance of the correlations, and the Kaiser-Meyer-Olkin (KMO) test, which indicates the proportion of variance in the data (p < 0.05 and KMO > 0.50, indicative of adequacy, respectively)^12^.

The number of factors was estimated using parallel analysis. This was followed by EFA with a tetrachoric correlation matrix, promax oblique rotation, and the unweighted least squares extraction method^12^.

The resulting model was jointly evaluated by factor loadings, communalities and the Tucker-Lewis fit index (TLI), root mean square error of approximation (RMSEA), and Bayesian information criterion (BIC). Factor loadings ≥ 0.30 and < 0.85 and communality > 0.20^14,15^ were accepted. In addition, it was recommended that there should be no double saturation or cross-loadings, i.e. items with a significant loading on different factors^12^. TLI values > 0.90, RMSEA < 0.08^14^and the lowest BIC value were considered adequate for deciding between the models.

Critical analysis of the form considered compliance with these fit parameters. To this end, some corrective actions were undertaken in this process, followed by re-specification and re-analysis of the model. The following actions were carried out: 1) evaluating variables for exclusion, considering their general contribution to the object analyzed, or for grouping, when there is a similarity between foods and food groups; and 2) changing the number of factors extracted^12^. The corrective actions took place sequentially, starting with the critical evaluation of the variables to propose exclusions or groupings. Inadequate factor loadings were then analyzed and, finally, the number of factors extracted was changed. All the EFA stages were carried out using the psych package in the R Studio software, version 4.2.1.

### Analysis of Measurement Invariance of Sisvan Food Intake Markers for Children Aged Six to 23 Months and 29 Days

After critically analyzing the form and understanding its internal structure, with satisfactory parameters, an analysis of invariance was carried out in three groups of interest: 1) in age groups (six to < 12 months, 12 to < 18 months, and 18 to < 24 months), to investigate the structure stability in the introduction and continuity of complementary feeding; 2) in the Brazilian macro-regions Midwest, Northeast, North, Southeast, and South, assessing invariance in the light of different feeding practices nationwide; and 3) longitudinally, from 2015 to 2019, looking at the invariance over the years. Multigroup confirmatory factor analysis (MCFA) was used for each section, stipulating the number and distribution of items in the factors,^12,13,16^ in three sequentially restrictive models:

- Model A - Configural invariance or equivalence of form: assesses the plausibility of the number of factors and items per factor;
- Model B - Metric invariance or equivalence of factor loadings: investigates the equivalence of the factor loadings of the items;
- Model C - Scalar invariance or equivalence of intercepts: examines the relationship between the scores obtained and the level of the latent trait.

Configural invariance was accepted when RMSEA < 0.08, TLI and comparative fit index (CFI) > 0.90^14^. Metric and scalar invariance were then tested and accepted when ΔRMSEA < 0.015 and ΔCFI < 0.01 when comparing models B and A and models C and B^16^. All the MCFA steps were carried out using the lavaan package in the R Studio software, version 4.2.1.

## RESULTS

**Figure 1:**
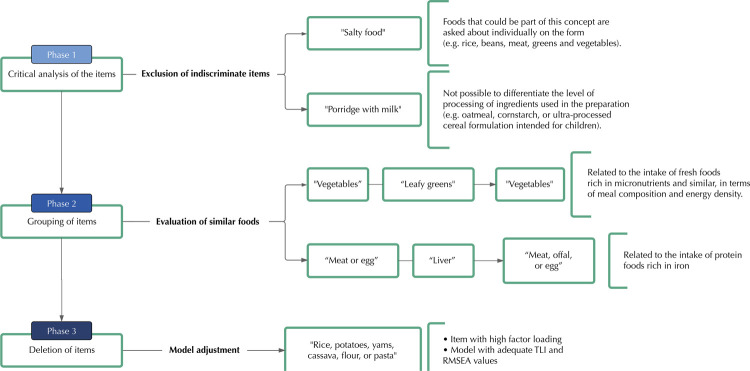
Flow of corrective actions for the Food and Nutrition Surveillance System (Sisvan) form of food intake markers for children aged six to 23 months and 29 days.

**Figure 2:**
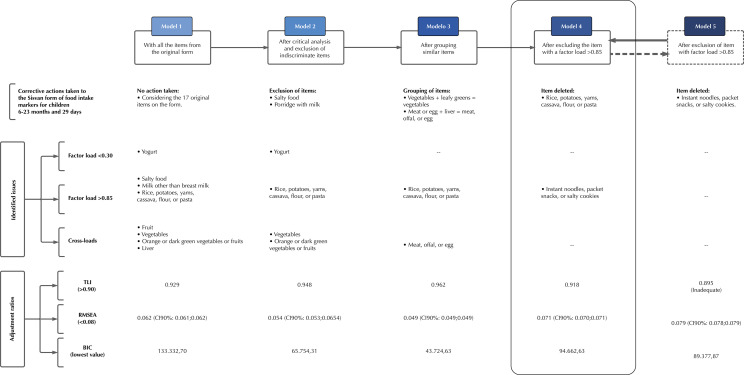
Presentation of the fit indices and problems of the exploratory factor analysis models of the Food and Nutrition Surveillance System (SISVAN) form of food intake markers for children aged six to 23 months and 29 days.

The sample had adequate parameters for the EFA (p < 0.001 in Bartlett’s test of sphericity and KMO = 0.81). The flow of corrective actions in the exploratory phase is shown in Figure 1, with the problems observed summarized in Figure 2.

RMSEA: root mean square error of approximation. TLI: Tucker-Lewis index.

RMSEA: root mean square error of approximation. TLI: Tucker-Lewis index. BIC: Bayesian information criterion.

Considering the 17 items on the form, the first model consisted of five factors and showed adequate TLI and RMSEA values. However, there were inadequate factor loadings and double saturation. A critical analysis of the items was carried out, excluding “salty food” and “porridge with milk”, which were considered non-specific to the object being assessed. It was assumed that the first item could cover a set of subsequent questions on the form, while the second did not make it possible to discriminate the level of processing of the preparation’s ingredients. After this action, model 2 showed adequate TLI and RMSEA values and a lower BIC value when compared to model 1, but with inadequate factor loadings and cross-loadings.

Similar items were grouped together, with “vegetables” and “leafy greens” making up the “vegetables” item, and “meat or egg” and “liver” making up the “meat, offal, or egg” item. Model 3 showed four factors and adequate TLI and RMSEA values, but high factor loadings and double saturation remained.

The next corrective action discarded the item with a high factor loading (“rice, potatoes, yams, cassava, flour, or pasta”). Model 4 showed adequate TLI and RMSEA values, with no cross-loadings and one item with a factor loading above the parameters (“instant noodles, packet snacks, or salty cookies”: 0.87). Finally, this item was excluded, but the model 5 had an inadequate TLI value and a borderline RMSEA value, with inadequate fit.

Model 4 was therefore considered to be the most appropriate solution, accepting the item “instant noodles, packet snacks, or salty cookies” with a slightly higher factor load, recognizing its relevance to characterizing the latent trait of infant feeding practices. The final model had satisfactory fit statistics and consisted of three dimensions (factor 1 - ultra-processed food: yogurt, hamburgers and/or sausages, sweetened drinks, instant noodles, packet snacks, or salty cookies, and sandwich cookies, sweets, or candies; factor 2 - healthy complementary food: whole fruit, vegetables, orange or dark green leafy vegetables or fruits, meat, offal, or egg, and beans; factor 3 - milk feeding: breast milk and milk other than breast milk), as illustrated in Table 1.

**Table 1:** Description of the factor loadings, communalities, variances, and fit indices of the three dimensional exploratory model for the Food and Nutrition Surveillance System (Sisvan) form of food intake markers for children aged six to 23 months and 29 days.

Food intake markers	Factor loading^b^			h^2^

	Factor 1	Factor 2	Factor 3	
Breast milk	-	-	0.80	0.58
Milk other than breast milk	-	-	0.59	0.34
Whole fruit	-	0.66	-	0.38
Vegetables (vegetables or leafy greens)	-	0.79	-	0.55
Orange or dark green leafy vegetables or fruits	-	0.79	-	0.54
Meat, offal, or egg	-	0.61	-	0.54
Beans	-	0.56	-	0.43
Yogurt	0.32	-	-	0.25
Hamburgers and/or sausages	0.72	-	-	0.50
Sweetened drinks	0.77	-	-	0.56
Instant noodles, packet snacks, or salty cookies	0.87	-	-	0.66
Sandwich cookies, sweets, or treats	0.82	-	-	0.64
Variance^a^	0.22	0.20	0.08	-
Fit indices^b^	-	-	-	-
RMSEA (90% CI)	0.071 (0.070;0.071)			
TLI	0.918			

Factor 1: ultra-processed food. Factor 2: healthy complementary feeding. Factor 3: milk feeding.

h^2^: communality. RMSEA: root mean square error of approximation. TLI: Tucker-Lewis index.

^a^Explained variance of each factor (normalized between 0 and 1).

^b^Significance parameters: factor loadings ≥ 0.30 and < 0.85. RMSEA < 0.08. TLI > 0.90.

Table 2 shows the results of the measurement invariance analyses. The configuration of the model, in terms of number of factors and items per factor, remained equivalent across age groups, macro-regions, and over the years from 2015 to 2019 (model A). Evidence of metric invariance (model B in relation to A) and scalar invariance (model C in relation to B) was also accepted in the three groups of interest, attesting to the stability of the factor loadings of the items and the level of latent trait for the three factors, respectively. The Box illustrates the reformulated proposal for the instrument.

**Table 2:** Evidence of measurement invariance for the Food and Nutrition Surveillance System (Sisvan) form of food intake markers for children aged six to 23 months and 29 days between age groups, macro-regions and years (2015-2019).

Groups of interest	Fit indices^a^					

	RMSEA (90% CI)	TLI	CFI	Comparison	ΔRMSEA	ΔCFI
Age groups						
A. Configural invariance	0.047 (0.047;0.047)	0.921	0.939	-	-	-
B. Metric invariance	0.044 (0.044;0.045)	0.931	0.940	B vs A	-0.003	0.001
C. Scalar invariance	0.047 (0.047;0.047)	0.922	0.935	C vs B	0.003	-0.005
Macro-regions						
A. Configural invariance	0.053 (0.053;0.053)	0.909	0.930	-	-	-
B. Metric invariance	0.048 (0.048;0.048)	0.926	0.935	B vs A	-0.005	0.005
C. Scalar invariance	0.053 (0.053;0.054)	0.909	0.923	C vs B	0.005	-0.012
Years (2015 to 2019)						
A. Configural invariance	0.053 (0.053;0.054)	0.913	0.933	-	-	-
B. Metric invariance	0.048 (0.048;0.048)	0.930	0.938	B vs A	-0.005	0.005
C. Scalar invariance	0.051 (0.051;0.052)	0.919	0.932	C vs B	0.003	-0.006

Age groups: six to < 12 months, 12 to < 18 months and 18 to < 24 months. Macro-regions: Midwest, Northeast, North, Southeast, and South. RMSEA: root mean square error of approximation. TLI: Tucker-Lewis index. CFI: comparative fit index. ΔRMSEA: difference between RMSEA values; ΔCFI: difference between CFI values (when comparing models B and A and models C and B).

^a^Significance parameters: TLI and CFI > 0.90. ΔRMSEA < 0.015. ΔCFI < 0.01.

**Box:** Reformulated proposal for the Food and Nutrition Surveillance System (Sisvan) form of food intake markers for children aged six to 23 months and 29 days.

On the previous day, the child was fed:			
Breast milk	○ Yes	○ No	○ Don’t know
Milk other than breast milk	○ Yes	○ No	○ Don’t know
Whole, chopped, or mashed fruit	○ Yes	○ No	○ Don’t know
Vegetables (vegetables or leafy greens)	○ Yes	○ No	○ Don’t know
Orange vegetables or fruits (pumpkin or jerimum, carrots, papaya, mango) or dark green leaves (cabbage, kale, purslane, Jerusalem artichoke, spinach, mustard greens)	○ Yes	○ No	○ Don’t know
Meat, offal, or egg	○ Yes	○ No	○ Don’t know
Beans	○ Yes	○ No	○ Don’t know
Yogurt	○ Yes	○ No	○ Don’t know
Hamburger and/or sausages (ham, mortadella, salami, sausage)	○ Yes	○ No	○ Don’t know
Sweetened beverages (soda, boxed juice, powdered juice, boxed coconut water, guarana/ginger syrups, fruit juice with added sugar)	○ Yes	○ No	○ Don’t know
Instant noodles, packet snacks, or salty cookies	○ Yes	○ No	○ Don’t know
Sandwich cookies, sweets, or treats (candies, lollipops, chewing gum, caramel, jelly)	○ Yes	○ No	○ Don’t know
Rice, potatoes, yams, cassava, flour, or pasta (not instant noodles)*	○ Yes	○ No	○ Don’t know

*Recommendation to keep the item for evaluation of the indicator of minimum dietary diversity.

## DISCUSSION

This study brings together unprecedented evidence on the internal structure of the Sisvan form of food intake markers for children aged six to 23 months, as well as its invariance for measuring infant feeding practices. The findings confirmed the stability of the factors of healthy complementary feeding, ultra-processed foods, and milk feeding between age groups, Brazilian macro-regions, and longitudinally.

Internationally, there have been efforts to validate instruments for assessing food intake in the first years of life. In Australia, for example, the reliability and validity of a form that classifies the risk of inadequate nutrition in children aged 12 to 36 months, based on food intake in the previous week, has been evaluated^17^. In the United States, the correspondence between the quality of the diet and the consumption of 14 groups of specific foods and nutrients was analyzed, 10 of which related to the adequacy of the diet (fruits, vegetables, legumes, whole grains, dairy products, animal and vegetable protein sources, seafood, linoleic acid, and alpha-linoleic acid) and four related to the recommendation of moderate consumption (fruit juice, refined grains, sodium, and added sugars)^18^,.

In Brazil, Oliveira et al. proposed a form and 18 infant feeding indicators up to two years of age. The form was based on a theoretical model on the attributes, components and markers of complementary feeding^19^, to enable the evaluation of indicators recommended by the World Health Organization. However, although efforts were made to adapt the questions to the target audience, there were no assessments of the understanding of the structure, robustness, and validity of the instrument.

The unsatisfactory fit of the original version of the Sisvan form revealed a gap between the instrument’s expectation of objectively assess the phenomenon for which it was proposed and its actual measurement capacity. In the critical analysis that led to corrective actions, the focus was on items that were not specific to the object being assessed and the possibilities of grouping together those with similar characteristics. The internal structure benefited from the reformulation proposal, providing a more rational support for the professional routine of monitoring infant feeding practices in PHC services. The shorter and more precise resulting instrument is in line with the challenges of organizing work in the SUS.

The corrective actions did not affect the estimation of infant feeding indicators. The items maintained with statistical support are satisfactory for constructing the main indicators recommended by international bodies for this age group^20^. With regard to the exclusion of the item “rice, potatoes, yams, cassava, flour, or pasta (other than instant noodles)”, it should be considered that, as a Brazilian staple food group, the intake of these foods does not particularly differentiate the quality of the diet, which is a characteristic expected of a food intake marker^21^. However, as this group is part of international indicators of minimum dietary diversity for children aged six to 23 months^20^, its maintenance in the form is viable (Box) to enable the composition of globally comparable metrics with Sisvan records, although without contributing to the measurement of factors.

After critical analysis, the Sisvan food intake markers covered three crucial factors for dietary recommendations at this stage of life. It is known that the diversity and combination of food groups are important to provide nutritional balance and the evolution of food consistency according to child development^20,22^. On the other hand, a diet with excessive amounts of calories, salt, sugar, fats, and additives and especially ultra-processed foods is not recommended up to the age of two. In addition to the nutritional imbalance, these dietary components can reduce children’s interest in fresh foods, de-characterize main meals, and increase the risk of chronic non-communicable diseases^2,20,22^. The factors healthy complementary feeding and milk feeding were in line with dietary recommendations for this age group, while the ultra-processed food factor summarized dietary inadequacies in relation to current guidelines.^2,20^

It is also worth noting that the factors underlying the food intake markers for children aged six to 23 months are linked to recent investigations into the dietary patterns of this population group in Brazil. Using representative data from the National Health Survey (2013), Flores et al. identified three patterns in the dietary intake of children up to 23 months: 1) healthy foods (fruit, vegetables, meat and eggs, potatoes/cassava, cereals, cookies, and beans); 2) milk (breast milk, other milk, and porridge); and 3) unhealthy foods (sweets, soft drinks, and artificial juices)^23^. Carvalho et al., in turn, observed two patterns for children aged six to 23 months: 1) minimally processed, characterized by the consumption of non-breast milk or dairy products, fruit or natural juice, vegetables, beans or other legumes, meat or eggs, potatoes and other tubers and roots, and cereals and dairy products; and 2) ultra-processed, consisting of the consumption of artificial juices, sweets, candies or other foods with sugar, and soft drinks^24^. These findings corroborate that the internal structure of the Sisvan form of food intake markers reflects key points in the diet of children aged six to 23 months.

According to the MCFA, the invariance of items, factor loadings and latent trait scores between age groups, Brazilian macro-regions and longitudinally was confirmed. These findings are supported by factors that denoted a transition in the consistency and composition of the diet, as one factor made up of liquid dairy items and two factors made up of a set of pasty, semi-solid, and solid foods^2^. It can therefore be assumed that the items in the instrument adequately cover age groups in the successive phases of complementary feeding.

Our finding also suggested that the Sisvan food intake markers bring together questions about infant feeding practices that have capillarity in multiple scenarios in which PHC services are inserted among the Brazilian macro-regions, also capturing dietary characteristics that have remained relevant in recent years. According to the Brazilian Household Budged Survey (POF) 2017-2018, 48.7% of the total calories available in the household came from fresh and minimally processed foods and 19.4% from ultra-processed foods, varying from 11.9% in the North to 23.5% in the South. Compared to data from the 2002-2003 POF, in 2017-2018 there was an increasing trend in the acquisition and the share of ultra-processed foods in total calories consumed (+0.31 percentage points/year), to the detriment of fresh and minimally processed foods (-0.15 percentage points/year)^25^.

Although the POF does not directly assess the food intake of children aged six to 23 months, the relative share of food via household acquisition can influence individual infant feeding practices. According to the 2015 Pelotas (RS) birth cohort, the ultra-processed foods most consumed by children aged six to 23 months were: yogurt (88.3%), boxed juice, powdered juice or coconut water (65.8%), sweet or sandwich cookies (64.5%), candies, lollipops, chocolate, or jelly (64.4%), packet snacks (46.0%), breaded chicken, hamburger, or ultra-processed meats (43.1%), chocolate milk (43.0%), soft drinks (37.4%), and instant noodles (29.6%)^26^. Similarly, the MINA-Brazil birth cohort, in the northern region of the country, observed frequent consumption of: cookies (66.4%), industrialized yogurt (53.2%), sweets (18.1%), packet snacks (17.9%), and artificial juice (14.7%) at 12 months of age^27^.

In children aged six to 23 months, Enani-2019 revealed, for example, a low prevalence of intake of food rich in vitamin A (38.6%), along with a high consumption of sweetened drinks (24.5%), with no significant differences between macro-regions^8^. This diffuse panorama of dietary inadequacies supports the uniformity of the measurement characteristics of Sisvan food intake markers in the age group investigated between Brazilian macro-regions and longitudinally.

The results of this study should be interpreted considering its limitations. As this was an analysis of a form that had already been implemented nationally for FNS actions, it was possible to explore evidence of validity and invariance of the instrument’s internal structure, without carrying out primary analyses of content validity and the item response process. The record of Sisvan food intake markers has low population coverage^28^ but the sample size fully satisfied all requirements for psychometric analyses. Among the strengths are the availability of nationwide data, with responses to the form in situations for which it was designed, from the PHC services of the SUS.

This study joins other efforts to validate the Sisvan food intake markers^29^. Evidence can contribute to the dissemination of the use of the form in epidemiological research and population surveys among children from six to 23 months, as an instrument with consistent results in terms of its internal structure and measurement equivalence in the groups analyzed. The results of this study shall also be integrated into the expanded concept of FNS, as recommended by the Brazilian National Food and Nutrition Policy^30^, while strengthening the analysis of data produced continuously by health workers and the systematic food and nutrition monitoring, especially for children under two years of age.

## CONCLUSION

After critical analysis and corrective actions, the Sisvan food intake markers for children aged six to 23 months reflected crucial aspects of infant feeding practices at this stage of life. The three complementary feeding factors identified are linked to current recommendations for health promotion from the earliest years and showed stability in configuration, factor loadings, and scores between age groups, Brazilian macro-regions, and longitudinally. The internal structure and evidence of invariance in the reformulated version of the instrument support the use of the markers to monitor food intake during complementary feeding.
