# Supplementary material for: Sisvan food intake markers from six to 23 months: critical analysis and validation
Source: Rev Saude Publica. 2024 Jul 22;58:35. doi: 10.11606/s1518-8787.2024058005811 (PMC11319038; doi:10.11606/s1518-8787.2024058005811)
Supplement: Translation

# Translation: Marcadores do consumo alimentar do Sisvan de seis a 23 meses: análise crítica e validação

**Authors:** Bianca de Melo Guedes, Thanise Sabrina Souza Santos, Paula dos Santos Leffa, Sara Araújo da Silva, Bárbara Hatzlhoffer Lourenço

**Affiliations:** I Brasil Universidade de São Paulo. Faculdade de Saúde Pública. Programa de Pós-Graduação Nutrição em Saúde Pública. São Paulo, SP, Brasil; II Brasil Universidade de São Paulo. Faculdade de Saúde Pública. Núcleo de Pesquisas Epidemiológicas em Nutrição e Saúde. São Paulo, SP, Brasil; III Brasil Ministério da Saúde. Coordenação de Gestão de Projetos de Pesquisa. Brasília, DF, Brasil; IV Brasil Ministério da Saúde. Coordenação-Geral de Alimentação e Nutrição. Brasília, DF, Brasil; V Brasil Universidade de São Paulo. Faculdade de Saúde Pública. Departamento de Nutrição. São Paulo, SP, Brasil

**Keywords:** Vigilância Alimentar e Nutricional, Ingestão de Alimentos, Alimentação Complementar, Lactente, Estudo de Validação

## Abstract

**OBJETIVOS:**

Explorar a estrutura interna e analisar evidências de invariância de mensuração do formulário de marcadores do consumo alimentar do Sistema de Vigilância Alimentar e Nutricional (Sisvan) para crianças de seis a 23 meses.

**MÉTODOS:**

Utilizaram-se microdados do Sisvan de 2015 a 2019. Após análise de adequação da amostra, realizou-se análise fatorial (AF) exploratória considerando valores de cargas fatoriais (≥ 0,30 e < 0,85), comunalidade (> 0,20) e índices de ajuste do modelo – índice de Tucker-Lewis (TLI) > 0,90 e raiz do erro quadrático médio de aproximação (RMSEA) < 0,08. Empreendeu-se uma análise crítica aos itens do formulário para adequação dos parâmetros. A estabilidade de mensuração foi investigada entre grupos etários, macrorregiões e longitudinalmente por AF confirmatória multigrupo em modelos de invariância configural, métrica e escalar. A invariância configural foi aceita se RMSEA < 0,08 e TLI e índice de ajuste comparativo (CFI) > 0,90. Invariâncias métrica e escalar foram aceitas se ΔRMSEA < 0,015 e ΔCFI < 0,01, comparando-se ao modelo anterior.

**RESULTADOS:**

Após análise crítica, itens do formulário foram agrupados (legumes e verduras; carnes ou ovos e fígado) e excluídos (comida de sal; mingau com leite; arroz, batata, inhame, mandioca, farinha ou macarrão). O modelo exploratório compreendeu três fatores: alimentação complementar saudável (frutas; hortaliças; vegetal ou fruta alaranjados ou folhas verdes escuras; carnes, vísceras ou ovo; feijão), ultraprocessada (iogurte; hambúrguer e/ou embutidos; bebidas adoçadas; macarrão instantâneo, salgadinhos de pacote ou biscoitos salgados; biscoito recheado, doces ou guloseimas), e láctea (leite do peito; outro leite), com cargas fatoriais, comunalidades e ajuste satisfatórios (TLI: 0,918, RMSEA: 0,071). O instrumento reformulado foi estável nos modelos de invariância testados.

**CONCLUSÕES:**

Com a incorporação das modificações ao formulário do Sisvan, os marcadores subsidiam uma interpretação mais adequada da situação alimentar de crianças de seis a 23 meses.

## INTRODUÇÃO

Os dois primeiros anos de vida são cruciais para a promoção de hábitos saudáveis^1^. Entre seis e 23 meses, o início da alimentação complementar concomitante à continuidade do aleitamento materno é sensível à ocorrência de práticas alimentares inadequadas^2,3^. Segundo o Fundo das Nações Unidas para a Infância, a falta de diversidade, a baixa frequência alimentar e o consumo de alimentos ultraprocessados neste período apresentam riscos para o desenvolvimento de má nutrição^3^.

Pesquisas de demografia e saúde de 91 países de baixa e média renda constataram a ausência do consumo de frutas e hortaliças em 44,0% e consumo de carne ou ovo em 42,1% das crianças de seis a 23 meses^4^. Globalmente, apenas 27,1% apresentaram alimentação com a diversidade mínima de grupos alimentares (IC95%: 23,9–30,3) e 48,7% realizaram refeições na frequência mínima preconizada (IC95%: 45,2–52,2)^5^.

No Estudo Nacional de Alimentação e Nutrição Infantil (Enani-2019)^6,7^, observou-se prevalência elevada de consumo de alimentos ultraprocessados (80,5%) e de exposição ao açúcar (68,4%) na faixa etária de seis a 23 meses, concomitante à ausência do consumo de frutas e hortaliças em 22,2% das crianças^8^. Evidencia-se, assim, a necessidade de acompanhamento sistemático da alimentação infantil.

No Brasil, a vigilância alimentar e nutricional (VAN), no âmbito da atenção primária à saúde (APS) do Sistema Único de Saúde (SUS), abrange a avaliação de marcadores do consumo alimentar. Para as crianças de seis a 23 meses, o formulário do Sistema de Vigilância Alimentar e Nutricional (Sisvan) tem foco na qualidade dos alimentos e no risco de carências nutricionais e excesso de peso^9^.

Embora a utilização dos marcadores do Sisvan seja recomendada desde 2015, não há evidências de validade do instrumento, especialmente sobre sua estrutura interna, à luz de recomendações alimentares para essa faixa etária. Além disso, considerando a amplitude de uso do formulário, não há evidências que sustentem a comparabilidade das informações nos grupos etários, dadas as sucessivas fases da introdução alimentar dos seis aos 23 meses, e entre os diferentes contextos alimentares no país. Desconhece-se, também, a estabilidade de mensuração dos marcadores ao longo dos anos, relevante às variações temporais das práticas alimentares da população.

Assim, o presente estudo objetivou explorar a estrutura interna do formulário de marcadores do consumo alimentar do Sisvan para crianças de seis a 23 meses e analisar evidências de invariância de mensuração entre grupos etários, macrorregiões brasileiras e longitudinalmente, de 2015 a 2019.

## MÉTODOS

### Marcadores do Consumo Alimentar do Sisvan para Crianças de Seis a 23 Meses e 29 Dias

O formulário de marcadores do consumo alimentar do Sisvan para crianças de seis a 23 meses e 29 dias reúne 20 questões referentes ao dia anterior^9^. Para o presente estudo, foram considerados 17 itens sobre o consumo de alimentos e/ou grupos alimentares: “leite do peito”, “fruta inteira, em pedaço ou amassada”, “comida de sal”, “outro leite que não o leite do peito”, “mingau com leite”, “iogurte”, “legumes”, “vegetal ou fruta de cor alaranjada ou folhas verdes escuras”, “verdura de folha”, “carne ou ovo”, “fígado”, “feijão”, “arroz, batata, inhame, aipim/macaxeira/mandioca, farinha ou macarrão”, “hambúrguer e/ou embutidos”, “bebidas adoçadas”, “macarrão instantâneo, salgadinhos de pacote ou biscoitos salgados” e “biscoito recheado, doces ou guloseimas”.

### Cessão e Gerenciamento de Dados

A Coordenação-Geral de Alimentação e Nutrição (CGAN) cedeu os microdados do Sisvan do período de 2015 a 2019, conforme Portaria n° 884/2011 do Ministério da Saúde^10^e observada a aprovação do estudo pelo Comitê de Ética em Pesquisa da Faculdade de Saúde Pública da Universidade de São Paulo (parecer n° 4.172.787). No gerenciamento dos dados, os registros foram examinados segundo: número de identificação, sexo, data de nascimento, código do município e data de acompanhamento, para exclusão de ocorrências repetidas. Em casos de múltiplos registros do mesmo indivíduo em única data de acompanhamento (10,35% das observações), manteve-se o último registro do dia. Subtraíram-se os registros de indivíduos com o mesmo número de identificação, provenientes de municípios distintos (0,44% das observações). Excluíram-se as observações com idade inconsistente (0,13% das observações).

Para as análises realizadas, manteve-se apenas o primeiro registro do indivíduo, por ano de acompanhamento, evitando-se respostas correlacionadas em diversos pontos no tempo. Em consonância aos requisitos para as análises psicométricas realizadas, consideraram-se apenas os registros com respostas válidas (“sim” ou “não”) para todos os itens; respostas “não sabe” ou em branco equivaleram a 9,84% dos registros.

A amostra analítica compreendeu 576.034 registros de marcadores do consumo alimentar de crianças com seis a 23 meses e 29 dias entre 2015 e 2019. O gerenciamento dos dados foi realizado no *software* Stata, versão 13.

### Análise da Estrutura Interna dos Marcadores do Consumo Alimentar do Sisvan para Crianças de Seis a 23 Meses e 29 Dias

As evidências de validade de um instrumento classificam-se em cinco categorias: conteúdo, processo de resposta, estrutura interna, relação com outras variáveis e consequência de testagem^11^. O presente estudo ateve-se à análise da estrutura interna dos marcadores do consumo alimentar, realizada por meio da análise fatorial exploratória (AFE). Nesta técnica de interdependência, as variáveis agrupam-se em fatores que permitem identificar dimensões de avaliação latentes segundo as correlações entre elas, sem definição prévia da estrutura dos dados^12,13^. Como pré-requisito para a AFE, realizou-se a análise de adequação da amostra por meio do teste de esfericidade de Bartlett, que verifica a significância estatística geral das correlações, e do teste de Kaiser-Meyer-Olkin (KMO), que indica a proporção da variância dos dados (p < 0,05 e KMO > 0,50, indicativos de adequação, respectivamente)^12^.

A estimação do número de fatores ocorreu por meio da análise paralela. Sequencialmente, realizou-se a AFE com matriz de correlação tetracórica, rotação oblíqua promax e método de extração por mínimos quadrados não ponderados^12^.

O modelo resultante foi avaliado conjuntamente pelos valores de cargas fatoriais, comunalidades e dos índices de ajuste Tucker-Lewis (TLI), raiz do erro quadrático médio de aproximação (RMSEA) e critério de informação bayesiano (BIC). Aceitaram-se valores de cargas fatoriais ≥ 0,30 e < 0,85 e comunalidade > 0,20^14,15^. Adicionalmente, preconizou-se a não ocorrência de dupla saturação ou cargas cruzadas, isto é, itens com carga significante em mais um fator^12^. Consideraram-se adequados valores de TLI > 0,90, conjuntamente a RMSEA < 0,08^14^e menor valor de BIC para decisão entre os modelos.

A análise crítica do formulário considerou o atendimento a esses parâmetros de adequação. Para tanto, foram realizadas neste processo algumas ações corretivas, seguidas de reespecificação e reanálise do modelo. As seguintes ações foram operacionalizadas: 1) avaliação de variáveis para exclusão, considerando sua contribuição geral para o objeto analisado, ou para agrupamento, quando da semelhança entre alimentos e grupos alimentares; e 2) alteração do número de fatores extraídos^12^. As ações corretivas ocorreram de modo sequencial, a partir da avaliação crítica das variáveis para proposição de exclusões ou agrupamentos. Processualmente, foram analisadas cargas fatoriais inadequadas e, por fim, a alteração do número de fatores extraídos. Todas as etapas da AFE foram conduzidas com o pacote *psych* no *software* R Studio, versão 4.2.1.

### Análise de Invariância de Mensuração dos Marcadores do Consumo Alimentar do Sisvan para Crianças de Seis a 23 meses e 29 dias

Após análise crítica do formulário e compreensão da estrutura interna, com parâmetros satisfatórios, realizou-se a análise de invariância em três recortes de interesse: 1) em grupos etários (seis a < 12 meses, 12 a < 18 meses e 18 a < 24 meses), para investigar a manutenção da estrutura na introdução e continuidade da alimentação complementar; 2) nas macrorregiões Centro-Oeste, Nordeste, Norte, Sudeste e Sul, avaliando a invariância à luz de distintas práticas alimentares em âmbito nacional; e 3) longitudinalmente, de 2015 a 2019, averiguando a estabilidade da estrutura ao longo dos anos. Para cada recorte, empregou-se a análise fatorial confirmatória multigrupo (AFCMG), estipulando-se o número e a distribuição de itens nos fatores^12,13,16^, em três modelos sequencialmente restritivos:

- Modelo A - Invariância configural ou igualdade de forma: avalia a plausibilidade do número de fatores e itens por fator;
- Modelo B - Invariância métrica ou igualdade de cargas fatoriais: investiga a equivalência das cargas fatoriais dos itens;
- Modelo C - Invariância escalar ou igualdade de interceptos: examina a relação dos escores obtidos com o nível do traço latente.

Aceitou-se a invariância configural quando RMSEA < 0,08, TLI e índice de ajuste comparativo (CFI) > 0,90^14^. Sequencialmente, testaram-se as invariâncias métrica e escalar, que foram aceitas quando ΔRMSEA < 0,015 e ΔCFI < 0,01 na comparação dos modelos B e A e dos modelos C e B^16^. Todas as etapas da AFCMG foram conduzidas com o pacote *lavaan* no *software* R Studio, versão 4.2.1.

## RESULTADOS

A amostra apresentou parâmetros adequados para realização da AFE (p < 0,001 no teste de esfericidade de Bartlett e KMO = 0,81). O fluxo de ações corretivas na fase exploratória consta na Figura 1, com as problemáticas observadas resumidas na Figura 2.

**Figura 1:** Fluxo das ações corretivas realizadas para o conjunto de marcadores do consumo alimentar do Sistema de Vigilância Alimentar e Nutricional (Sisvan) para crianças de seis a 23 meses e 29 dias.

RMSEA: raiz do erro quadrático médio de aproximação. TLI: índice de Tucker-Lewis.

**Figura 2:** Apresentação dos índices de ajuste e das problemáticas dos modelos de análise fatorial exploratória do formulário de marcadores do consumo alimentar do Sistema de Vigilância Alimentar e Nutricional (Sisvan) para crianças de seis a 23 meses e 29 dias.

RMSEA: raiz do erro quadrático médio de aproximação. TLI: índice de Tucker-Lewis. BIC: critério de informação bayesiano.

Considerando os 17 itens do formulário, o primeiro modelo configurou-se em cinco fatores e apresentou valores adequados de TLI e RMSEA. Entretanto, observaram-se cargas fatoriais inadequadas e dupla saturação. Realizou-se a análise crítica dos itens, com exclusão de “comida de sal” e “mingau com leite”, considerados inespecíficos ao objeto avaliado. Compreendeu-se que o primeiro item poderia abarcar um conjunto de questões posteriores do formulário, enquanto o segundo não possibilitava discriminar o processamento dos ingredientes da preparação. Após essa ação, o modelo 2 apresentou valores adequados de TLI e RMSEA e menor valor de BIC, quando comparado ao modelo 1, porém com inadequações de cargas fatoriais e cargas cruzadas.

Procedeu-se ao agrupamento de itens similares, sendo que “legumes” e “verdura de folha” compuseram o item “hortaliças”, e “carne ou ovo” e “fígado”, o item “carnes, vísceras ou ovo”. O modelo 3 apresentou quatro fatores e valores adequados de TLI e RMSEA, mas mantiveram-se cargas fatoriais elevadas e dupla saturação.

A ação corretiva seguinte descartou o item com carga fatorial elevada (“arroz, batata, inhame, aipim/macaxeira/mandioca, farinha ou macarrão”). O modelo 4 apresentou valores adequados de TLI e RMSEA, sem cargas cruzadas e um item com carga fatorial acima dos parâmetros (“macarrão instantâneo, salgadinhos de pacote ou biscoitos salgados”: 0,87). Por fim, realizou-se a exclusão desse item, entretanto o modelo 5 obtido apresentou valor inadequado de TLI e valor limítrofe de RMSEA, com ajuste considerado inadequado.

Assim, considerou-se o modelo 4 como a solução mais adequada, aceitando-se o item “macarrão instantâneo, salgadinhos de pacote ou biscoitos salgados” com carga fatorial ligeiramente elevada ao reconhecer sua relevância à caracterização do traço latente de alimentação infantil nesta faixa etária. O modelo final teve estatísticas de ajuste satisfatórias e se configurou em três dimensões (fator 1 – alimentação ultraprocessada: iogurte, hambúrguer e/ou embutidos, bebidas adoçadas, macarrão instantâneo, salgadinhos de pacote ou biscoitos salgados, e biscoito recheado, doces ou guloseimas; fator 2 – alimentação complementar saudável: fruta inteira, hortaliças, vegetal ou fruta de cor alaranjada ou folhas verdes escuras, carnes, vísceras ou ovo, e feijão; fator 3 – alimentação láctea: leite do peito e outro leite que não o leite do peito), conforme ilustrado na Tabela 1.

**Tabela 1:** Descrição dos valores de cargas fatoriais, comunalidades, variâncias e índices de ajustes de modelo exploratório tridimensional para o formulário de marcadores do consumo alimentar do Sistema de Vigilância Alimentar e Nutricional (Sisvan) para crianças de seis a 23 meses e 29 dias.

Marcadores do consumo alimentar	Carga fatorial^b^			h^2^

	Fator 1	Fator 2	Fator 3	
Leite do peito	-	-	0,80	0,58
Outro leite que não do peito	-	-	0,59	0,34
Fruta inteira	-	0,66	-	0,38
Hortaliças (legumes ou verduras de folha)	-	0,79	-	0,55
Vegetal ou fruta de cor alaranjada ou folhas verdes escuras	-	0,79	-	0,54
Carnes, vísceras ou ovo	-	0,61	-	0,54
Feijão	-	0,56	-	0,43
Iogurte	0,32	-	-	0,25
Hambúrguer e/ou embutidos	0,72	-	-	0,50
Bebidas adoçadas	0,77	-	-	0,56
Macarrão instantâneo, salgadinhos de pacote ou biscoitos salgados	0,87	-	-	0,66
Biscoito recheado, doces ou guloseimas	0,82	-	-	0,64
Variância^a^	0,22	0,20	0,08	-
Índices de ajuste^b^	-	-	-	-
RMSEA (IC 90%)	0,071 (0,070;0,071)			
TLI	0,918			

Fator 1: alimentação ultraprocessada. Fator 2: alimentação complementar saudável. Fator 3: alimentação láctea.

h^2^: comunalidade. RMSEA: raiz do erro quadrático médio de aproximação. TLI: índice de Tucker-Lewis.

^a^Variância explicada de cada fator (normatizada entre 0 e 1).

^b^Parâmetros de significância: cargas fatoriais ≥ 0,30 e < 0,85. RMSEA < 0,08. TLI > 0,90.

A Tabela 2 apresenta os resultados das análises de invariância de mensuração. A configuração do modelo, em número de fatores e itens por fator, manteve equivalência nos grupos etários, macrorregiões e ao longo dos anos, de 2015 a 2019 (modelo A). As evidências de invariância métrica (modelo B em relação ao A) e escalar (modelo C em relação ao B) também foram aceitas nos três recortes investigados, atestando a estabilidade das cargas fatoriais dos itens e do nível de traço latente para os três fatores, respectivamente. O Quadro ilustra a proposta reformulada do instrumento.

**Tabela 2:** Evidências de invariância de mensuração para o conjunto de marcadores de consumo alimentar do Sistema de Vigilância Alimentar e Nutricional (Sisvan) para crianças de seis a 23 meses e 29 dias entre grupos etários, macrorregiões e anos (2015–2019).

Recortes de interesse	Índices de ajuste^a^					

	RMSEA (IC 90%)	TLI	CFI	Comparação	ΔRMSEA	ΔCFI
Grupos etários						
A. Invariância configural	0,047 (0,047;0,047)	0,921	0,939	-	-	-
B. Invariância métrica	0,044 (0,044;0,045)	0,931	0,940	B vs A	-0,003	0,001
C. Invariância escalar	0,047 (0,047;0,047)	0,922	0,935	C vs B	0,003	-0,005
Macrorregiões						
A. Invariância configural	0,053 (0,053;0,053)	0,909	0,930	-	-	-
B. Invariância métrica	0,048 (0,048;0,048)	0,926	0,935	B vs A	-0,005	0,005
C. Invariância escalar	0,053 (0,053;0,054)	0,909	0,923	C vs B	0,005	-0,012
Anos (2015 a 2019)						
A. Invariância configural	0,053 (0,053;0,054)	0,913	0,933	-	-	-
B. Invariância métrica	0,048 (0,048;0,048)	0,930	0,938	B vs A	-0,005	0,005
C. Invariância escalar	0,051 (0,051;0,052)	0,919	0,932	C vs B	0,003	-0,006

Grupos etários: seis a < 12 meses, 12 a < 18 meses e 18 a < 24 meses. Macrorregiões: Centro-Oeste, Nordeste, Norte, Sudeste e Sul. RMSEA: raiz do erro quadrático médio de aproximação. TLI: índice de Tucker-Lewis. CFI: índice de ajuste comparativo. ΔRMSEA: diferença entre valores de RMSEA; ΔCFI: diferença entre valores de CFI (na comparação dos modelos B e A e dos modelos C e B).

^a^Parâmetros de significância: TLI e CFI > 0,90. ΔRMSEA < 0,015. ΔCFI < 0,01.

**Quadro:** Proposta reformulada do formulário de marcadores do consumo alimentar do Sistema de Vigilância Alimentar e Nutricional (Sisvan) para crianças de seis a 23 meses e 29 dias.

Ontem a criança consumiu:			
Leite do peito	○ Sim	○ Não	○ Não sabe
Outro leite que não o leite do peito	○ Sim	○ Não	○ Não sabe
Fruta inteira, em pedaço ou amassada	○ Sim	○ Não	○ Não sabe
Hortaliças (legumes ou verdura de folha)	○ Sim	○ Não	○ Não sabe
Vegetal ou fruta de cor alaranjada (abóbora ou jerimum, cenoura, mamão, manga) ou folhas verdes escuras (couve, caruru, beldroega, bertalha, espinafre, mostarda)	○ Sim	○ Não	○ Não sabe
Carnes, vísceras ou ovo	○ Sim	○ Não	○ Não sabe
Feijão	○ Sim	○ Não	○ Não sabe
Iogurte	○ Sim	○ Não	○ Não sabe
Hambúrguer e/ou embutidos (presunto, mortadela, salame, linguiça, salsicha)	○ Sim	○ Não	○ Não sabe
Bebidas adoçadas (refrigerante, suco de caixinha, suco em pó, água de coco de caixinha, xaropes de guaraná/groselha, suco de fruta com adição de açúcar)	○ Sim	○ Não	○ Não sabe
Macarrão instantâneo, salgadinhos de pacote ou biscoitos salgados	○ Sim	○ Não	○ Não sabe
Biscoito recheado, doces ou guloseimas (balas, pirulitos, chiclete, caramelo, gelatina)	○ Sim	○ Não	○ Não sabe
Arroz, batata, inhame, aipim/macaxeira/mandioca, farinha ou macarrão (sem ser instantâneo)*	○ Sim	○ Não	○ Não sabe

*Recomendação de manutenção do item para a avaliação do indicador de diversidade alimentar mínima.

## DISCUSSÃO

Este estudo reúne evidências inéditas sobre a estrutura interna do formulário de marcadores do consumo alimentar do Sisvan para crianças de seis a 23 meses, bem como sua invariância para mensuração da alimentação infantil. Os achados confirmaram a estabilidade dos fatores de alimentação complementar saudável, alimentação ultraprocessada e alimentação láctea entre grupos etários, macrorregiões brasileiras e longitudinalmente.

Internacionalmente, observam-se esforços de validade de instrumentos para avaliação do consumo alimentar nos primeiros anos de vida. Na Austrália, por exemplo, avaliaram-se a confiabilidade e a validade de um formulário que classifica o risco de alimentação inadequada em crianças de 12 a 36 meses, a partir da ingestão de alimentos na semana anterior^17^. Nos Estados Unidos, analisou-se a correspondência entre a qualidade da dieta e o consumo de 14 grupos de alimentos e nutrientes específicos, sendo 10 grupos relativos à adequação da dieta (frutas, legumes e verduras, leguminosas, cereais integrais, laticínios, alimentos fonte de proteína animal e vegetal, frutos do mar, ácido linoleico e ácido alfalinoleico) e quatro referentes à recomendação de consumo moderado (suco de fruta, grãos refinados, sódio e açúcares de adição)^18^.

No Brasil, Oliveira et al. propuseram um formulário e 18 indicadores da alimentação até dois anos de vida. O formulário baseou-se em modelo teórico sobre atributos, componentes e marcadores da alimentação complementar^19^, para habilitar a avaliação de indicadores recomendados pela Organização Mundial da Saúde. Entretanto, embora tenham empreendidos esforços de adequação das questões ao público-alvo, não foram realizadas avaliações quanto à compreensão de estrutura, robustez e validade do instrumento.

O ajuste insatisfatório da versão original do formulário do Sisvan evidenciou um hiato entre a expectativa do instrumento em apreender objetivamente o fenômeno para o qual foi proposto e a sua real capacidade de mensuração. Na análise crítica que embasou ações corretivas, focalizaram-se itens inespecíficos ao objeto avaliado e possibilidades de agrupamento daqueles com características semelhantes. A estrutura interna foi beneficiada com a proposta de reformulação, subsidiando mais racionalmente a rotina profissional de acompanhamento das práticas alimentares infantis nos serviços da APS, com um instrumento mais enxuto e preciso, o que é convergente aos desafios da organização do trabalho no SUS.

As ações corretivas não acarretaram prejuízos ao cálculo dos indicadores de alimentação infantil. Os itens mantidos com respaldo estatístico são satisfatórios para a construção dos principais indicadores preconizados por órgãos internacionais para essa faixa etária^20^. Com relação à exclusão do item “arroz, batata, inhame, aipim/macaxeira/mandioca, farinha ou macarrão (sem ser instantâneo)” para a compreensão da estrutura interna do formulário, deve-se considerar que, como parte da base alimentar brasileira, o consumo destes alimentos não diferencia particularmente a qualidade da alimentação, característica esperada de um marcador do consumo^21^. Porém, como este grupo compõe indicadores internacionais de diversidade alimentar mínima para crianças de seis a 23 meses^20^, pode-se indicar sua manutenção (Quadro), ainda que sem contribuição para a mensuração dos fatores, para habilitar a composição de métricas comparáveis globalmente com os registros do Sisvan.

Após análise crítica, os marcadores do Sisvan abrangeram três fatores cruciais alinhados às recomendações alimentares nessa fase do curso da vida. Sabe-se que a diversidade e a combinação de grupos alimentares são importantes para prover o equilíbrio nutricional e a evolução de consistência dos alimentos conforme aspectos do desenvolvimento infantil^20,22^. Em contrapartida, alimentos com quantidades excessivas de calorias, sal, açúcar, gorduras e aditivos e, principalmente, alimentos ultraprocessados não são recomendados até os dois anos de idade, pois, além do desbalanço nutricional, podem reduzir o interesse das crianças por alimentos *in natura*, descaracterizar refeições principais e aumentar riscos para doenças crônicas não transmissíveis^2,20,22^. Os fatores alimentação complementar saudável e alimentação láctea alinharam-se à recomendação alimentar para essa faixa etária, enquanto o fator alimentação ultraprocessada sintetizou inadequações alimentares ante as orientações vigentes^2,20^.

Destaca-se, ainda, que os fatores subjacentes aos marcadores do consumo alimentar para crianças de seis a 23 meses conectam-se a investigações recentes dos padrões de consumo alimentar deste grupo populacional no Brasil. Usando dados representativos da Pesquisa Nacional de Saúde (2013), Flores et al. identificaram três padrões na ingestão alimentar de crianças até 23 meses: 1) alimentos saudáveis (frutas, legumes e verduras, carnes e ovos, batata/mandioca, cereais, bolachas e feijão); 2) leites (leite materno, outros leites e mingau); e 3) alimentos não saudáveis (doces, refrigerantes e sucos artificiais)^23^. Carvalho et al., por sua vez, observaram dois padrões para a faixa etária de seis a 23 meses: 1) minimamente processado, caracterizado pelo consumo de leite não materno ou derivados, frutas ou suco natural, verduras/legumes, feijão ou outras leguminosas, carnes ou ovos, batata e outros tubérculos e raízes, e cereais e derivados; e 2) ultraprocessado, constituído pelo consumo de sucos artificiais, doces, balas ou outros alimentos com açúcar e refrigerantes^24^. Estes achados corroboram que a estrutura interna do formulário de marcadores do consumo alimentar do Sisvan reflete pontos centrais na alimentação das crianças de seis a 23 meses.

Segundo a AFCMG, confirmou-se a invariância de itens, cargas fatoriais e escores do traço latente entre grupos etários, macrorregiões brasileiras e longitudinalmente. Esses achados se sustentam considerando que os fatores identificados denotaram transição de consistência e composição da alimentação, ao delimitarem um fator composto por itens lácteos líquidos e dois fatores compostos por um conjunto de alimentos pastosos, semissólidos e sólidos^2^. Pode-se assumir, portanto, que os itens do instrumento cobrem adequadamente grupos etários das sucessivas fases da alimentação complementar.

Pode-se admitir, ainda, que os marcadores do Sisvan reúnem perguntas sobre consumo alimentar infantil que têm capilaridade em múltiplos cenários em que se inserem os serviços da APS entre as macrorregiões brasileiras, captando, ainda, características alimentares que se mantiveram relevantes nos últimos anos. Segundo a Pesquisa de Orçamentos Familiares (POF) 2017–2018, do total de calorias disponíveis no domicílio, 48,7% foram provenientes de alimentos *in natura* e minimamente processados e 19,4%, de alimentos ultraprocessados, cuja presença variou de 11,9% na região Norte a 23,5% na região Sul. Comparativamente aos dados da POF 2002–2003, em 2017–2018, houve tendência de aumento da aquisição e participação calórica de alimentos ultraprocessados (+0,31 pontos percentuais/ano), em detrimento de alimentos *in natura* e minimamente processados (-0,15 pontos percentuais/ano)^25^.

Embora a POF não avalie diretamente o consumo alimentar das crianças de seis a 23 meses, a participação relativa dos alimentos via aquisição domiciliar pode influenciar o consumo alimentar individual infantil. De acordo com a coorte de nascimentos de Pelotas (RS) de 2015, os alimentos ultraprocessados mais consumidos por crianças de seis a 23 meses foram: iogurte (88,3%), suco de caixinha, em pó ou água de coco (65,8%), biscoitos doces ou recheados (64,5%), balas, pirulitos, chocolate ou geleia (64,4%), salgadinhos de pacote (46,0%), empanado de frango, hambúrguer ou carnes ultraprocessadas (43,1%), achocolatado (43,0%), refrigerantes (37,4%) e macarrão instantâneo (29,6%)^26^. Similarmente, a coorte de nascimentos MINA-Brasil, na região Norte do país, observou consumo frequente de: biscoitos (66,4%), iogurte industrializado (53,2%), guloseimas (18,1%), salgadinhos de pacote (17,9%) e suco artificial (14,7%) aos 12 meses^27^.

Em crianças de seis a 23 meses, o Enani-2019 revelou, por exemplo, baixa prevalência de consumo de alimentos fontes de vitamina A (38,6%), paralelamente ao elevado consumo de bebidas adoçadas (24,5%), sem diferenças significativas entre macrorregiões^8^. Este panorama difuso de inadequações alimentares sustenta a uniformidade das características de mensuração dos marcadores do consumo alimentar do Sisvan na faixa etária investigada entre macrorregiões brasileiras e longitudinalmente.

Os resultados do presente estudo devem ser interpretados considerando suas limitações. Tratando-se da análise de um formulário já implementado nacionalmente para as ações de VAN, foi possível explorar evidências de validade e invariância da estrutura interna do instrumento, sem a realização de análises primárias de validade de conteúdo e processo de resposta dos itens. O registro de marcadores do consumo alimentar do Sisvan tem baixa cobertura populacional^28^, mas o tamanho amostral satisfez plenamente as análises psicométricas realizadas. Entre pontos fortes, salientam-se a abrangência nacional dos dados e a disponibilidade de respostas aos formulários em situações para as quais este foi idealizado, a partir dos serviços da APS do SUS.

O presente estudo soma-se a outros esforços para validação dos marcadores do consumo alimentar do Sisvan^29^e espera-se que as suas evidências possam contribuir para a disseminação do uso do formulário em pesquisas epidemiológicas e recortes populacionais para a fase dos seis aos 23 meses, como um instrumento com resultados consistentes quanto à sua estrutura interna e equivalência de mensuração nos grupos analisados. Almeja-se que os resultados deste estudo possam integrar o conceito ampliado de VAN preconizado pela Política Nacional de Alimentação e Nutrição^30^, com o fortalecimento da análise de dados produzidos continuamente por trabalhadores de saúde e o acompanhamento sistemático da situação alimentar nacional, em especial dos menores de dois anos.

## CONCLUSÃO

Após a análise crítica e os ajustes fundamentados, os marcadores do consumo alimentar do Sisvan para crianças de seis a 23 meses refletiram aspectos cruciais da alimentação nessa fase do curso da vida. Os três fatores de alimentação complementar identificados conectam-se a recomendações vigentes para promoção da saúde desde os primeiros anos e demonstraram estabilidade em configuração, cargas fatoriais e escore entre os grupos etários, macrorregiões brasileiras e longitudinalmente. A estrutura interna e as evidências de invariância na versão reformulada do instrumento subsidiam o uso dos marcadores para monitoramento do consumo alimentar na fase de alimentação complementar.
